# The Renin-Angiotensin System as a Component of Biotrauma in Acute Respiratory Distress Syndrome

**DOI:** 10.3389/fphys.2021.806062

**Published:** 2022-04-13

**Authors:** Katharina Krenn, Verena Tretter, Felix Kraft, Roman Ullrich

**Affiliations:** Department of Anesthesia, General Intensive Care and Pain Medicine, Medical University of Vienna, Vienna, Austria

**Keywords:** acute respiratory distress syndrome, ventilator-induced lung injury, renin-angiotensin system, biotrauma, mass spectrometry

## Abstract

Acute respiratory distress syndrome (ARDS) is a major concern in critical care medicine with a high mortality of over 30%. Injury to the lungs is caused not only by underlying pathological conditions such as pneumonia, sepsis, or trauma, but also by ventilator-induced lung injury (VILI) resulting from high positive pressure levels and a high inspiratory oxygen fraction. Apart from mechanical factors that stress the lungs with a specific physical power and cause volutrauma and barotrauma, it is increasingly recognized that lung injury is further aggravated by biological mediators. The COVID-19 pandemic has led to increased interest in the role of the renin-angiotensin system (RAS) in the context of ARDS, as the RAS enzyme angiotensin-converting enzyme 2 serves as the primary cell entry receptor for severe acute respiratory syndrome (SARS) coronavirus (CoV)-2. Even before this pandemic, studies have documented the involvement of the RAS in VILI and its dysregulation in clinical ARDS. In recent years, analytical tools for RAS investigation have made major advances based on the optimized precision and detail of mass spectrometry. Given that many clinical trials with pharmacological interventions in ARDS were negative, RAS-modifying drugs may represent an interesting starting point for novel therapeutic approaches. Results from animal models have highlighted the potential of RAS-modifying drugs to prevent VILI or treat ARDS. While these drugs have beneficial pulmonary effects, the best targets and application forms for intervention still have to be determined to avoid negative effects on the circulation in clinical settings.

## Introduction

**FIGURE 1:**
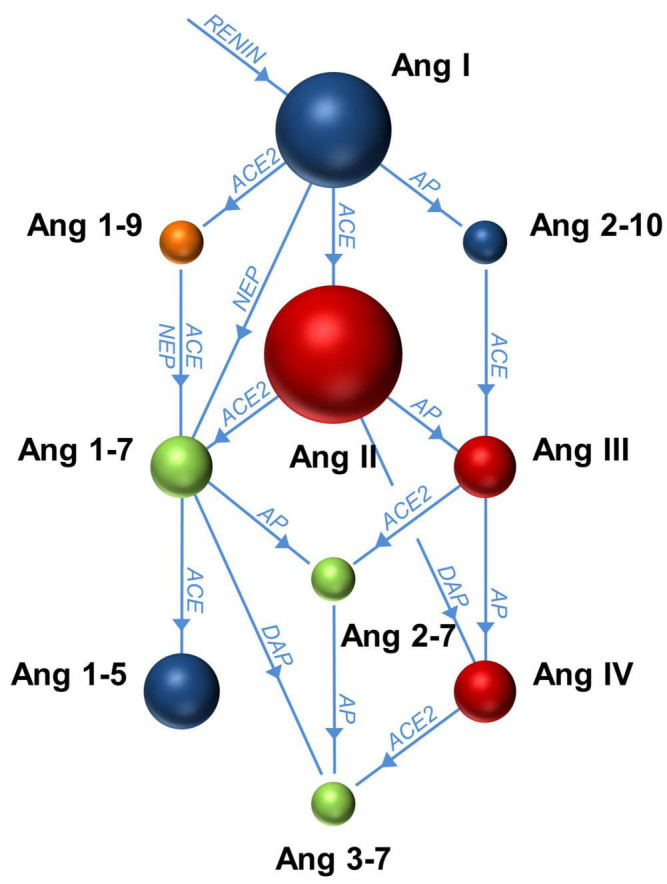
The network of enzymes and peptides of the renin-angiotensin system in plasma. The enzymatic cascade is initiated by cleavage of angiotensinogen by renin to produce angiotensin (Ang) I. The network of enzymes that successively cleave the respective angiotensin metabolites is indicated in blue. ACE, angiotensin-converting enzyme; AP, aminopeptidase; NEP, neprilysin; DAP, diaminopeptidase.

The renin-angiotensin system (RAS) plays a role in many cardiovascular, renal, and pulmonary processes. It is a network of peptides (Figure 1) that are enzymatically cleaved from the precursor protein angiotensinogen (56.8 kDa) that is mainly produced by the liver. The first step of RAS activation is the cleavage of angiotensinogen to angiotensin I (Ang I or Ang 1–10) by the protease renin, an enzyme produced by the kidney. In this context, “1–10” refers to the number of amino acid residues constituting the peptide. Ang I is subsequently cleaved to angiotensin II (Ang II or Ang 1–8) by angiotensin converting enzyme (ACE) (Skeggs et al., 1980; Corvol et al., 1995). Ang II-mediated effects are exerted through the Ang II type 1 (AT1) and 2 (AT2) receptors (Eckenstaler et al., 2021; Fatima et al., 2021). The production of Ang II by ACE is often called the “classical” activation of the RAS. Ang I and Ang II may also be enzymatically cleaved by other, “alternative” proteases including ACE2, resulting in a multitude of smaller peptides such as Ang 1–9 or Ang 1–7 (Imai et al., 2008; Santos et al., 2018). In addition to production of Ang 1–7 from Ang II by ACE2, Ang 1–7 may be cleaved from Ang II by prolyl oligopeptidase (POP) in the circulation (Serfozo et al., 2020). Another enzyme, neprilysin, a neutral endopeptidase, cleaves Ang I to Ang 1–7 (Rice et al., 2004). The peptide Ang 1–7 in turn is a substrate for the N-domain of ACE and cleaved to the smaller peptide Ang 1–5 (Corradi et al., 2006). In a broader context, there is complex interaction between the RAS, the kinin–kallikrein system and the activity of chymase, which is expressed by mast cells and various tissues and may also cleave Ang I to Ang II (Abassi et al., 2021). Renin, ACE and AT1 receptors are important targets of anti-hypertensive and heart failure therapy (Aronson and Krum, 2012; Laurent et al., 2012). Recently, a neprilysin inhibitor/angiotensin receptor blocker combination was added to the spectrum of heart failure therapy (Gallo et al., 2021). ACE inhibitors most importantly block the production of Ang II from Ang I and therefore lead to an accumulation of Ang I and subsequently the peptide Ang 2–10 that is cleaved from Ang I by aminopeptidase A (Velez et al., 2009), and Ang 1–7 that is cleaved from Ang I by neprilysin. As ACE inhibitors also block the degradation of Ang 1–7 to Ang 1–5, this leads to a further increase in Ang 1–7 levels that is detectable in the plasma of patients treated with ACE inhibitors (Kovarik et al., 2015, 2017). Angiotensin receptor blockers lead to increased levels of Ang II (Vischer et al., 2021), whereas inhibition of renin downregulates the concentrations of all angiotensin peptides, since the production of Ang I is already inhibited. This review describes the involvement of the RAS in the biological reactions to mechanical ventilation and summarizes what is known about RAS regulation in critically ill and mechanically ventilated patients with ARDS and COVID-19 for the purpose of discovering new biomarkers and identifying new therapeutic targets.

## Concepts of Ventilator-Induced Lung Injury

Mechanical ventilation is required in clinical conditions associated with acute pulmonary gas exchange deterioration, reduced vigilance, and loss of protective reflexes. The unphysiological state of positive pressure ventilation subsequently impacts the body further. The relevance of this impact increases with higher settings of mechanical power variables such as ventilation pressure, tidal volume and respiratory rate (Cressoni et al., 2016; Gattinoni et al., 2016). Components of ventilator-induced lung injury (VILI) include volutrauma and barotrauma, atelectrauma, and biotrauma (Gattinoni et al., 2010). Biotrauma involves upregulation of proinflammatory mediators and recruitment of neutrophils to the lungs which may augment pulmonary edema formation in VILI (Dreyfuss and Saumon, 1998). It is well established that experimental ventilation with high tidal volumes leads to increased levels of proinflammatory cytokines such as interleukin (IL)-6 and macrophage inhibitory protein (MIP)-2, a functional equivalent of IL-8 in rodents (Dreyfuss and Saumon, 1998; Halbertsma et al., 2005). This increase of proinflammatory mediators in the circulation caused by VILI may subsequently promote multiple system organ failure (Slutsky and Tremblay, 1998; Plotz et al., 2004). In a randomized controlled trial, Ranieri et al. (1999) showed that proinflammatory mediators in broncho-alveolar lavage fluid (BALF) and plasma were lower in a group of patients receiving lung-protective ventilation characterized by lower tidal volume and end-inspiratory plateau pressure as well as higher positive end-expiratory pressure (PEEP). The ARDS network study further underscored the clinical impact on ventilator-free days and survival of lung-protective ventilation with a reduced tidal volume of 6 ml/kg predicted body weight and an inspiratory plateau pressure of 30 cm of water at maximum (Acute Respiratory Distress Syndrome Network et al., 2000). At the interface of inflammatory activation and the fibroproliferative response that may occur following ARDS, scientists began to understand the relevance of Ang II as a mediator. Ang II is released from the lungs of patients suffering from ARDS (Wenz et al., 2000), and experimental data have shown its profibrotic effects in lung injury caused by bleomycin (Marshall et al., 2004). In the latter study, treatment with the ACE inhibitor ramipril or the AT1 receptor blocker losartan resulted in reduced pulmonary collagen deposition. Hypoxia as well as hyperoxia may occur in mechanically ventilated lungs – hypoxia for instance as a consequence of hypoventilated areas attributable to the primary lung disease, hyperoxia as a consequence of the high inspiratory fraction of oxygen needed for treatment of hypoxemic patients. Hypoxia as well as hyperoxia induce collagen production in human pulmonary fibroblasts (Lang et al., 2010; Liu et al., 2013), and may be involved in the development of fibrotic changes following ARDS.

## Experimental Evidence About the Role of the Renin-Angiotensin System in Lung Injury

The RAS and potential sites of intervention have been studied in various experimental settings, sometimes yielding conflicting results that depend on the exact parameters of the experimental models. Ventilation with a very high tidal volume of 40 ml/kg in rats led to increased protein content in BALF, pulmonary expression and serum levels of MIP-2, and lung tissue levels of Ang II after 4 h, while mRNA expression levels of ACE2 were decreased (Jerng et al., 2007). The effects on BALF protein levels, MIP-2 and ACE2 expression were absent in a group of rats ventilated with a low tidal volume (7 ml/kg) and were reversible with the ACE inhibitor captopril. Beneficial effects on BALF protein levels in the high tidal volume group were also achieved by AT1 or AT2 receptor blockers in this study. Furthermore, studies have shown protective effects of ACE and AT1 receptor inhibition in rodent models of lung injury (Lukkarinen et al., 2005; He et al., 2007; Shen et al., 2009). In addition to increased levels of proinflammatory markers, Ang II may lead to an imbalance in the expression of epithelial sodium channel (ENaC) subunits that inhibits alveolar fluid clearance and thereby promotes pulmonary edema (Deng et al., 2012a,b). ACE2 is a natural counterregulator of classical RAS activation and the subsequent increase in Ang II levels. This homolog of ACE protects from ARDS, whereas increased ACE activity and signaling through AT1 receptors further aggravate lung injury (Imai et al., 2007). ACE2 inactivates Ang II and thus counteracts the deleterious effects of Ang II-signaling through AT1 receptors such as increased vascular tone and permeability (Imai et al., 2005, 2008). Mice with genetic inactivation of ACE were protected from acid-induced lung injury and revealed decreased Ang II levels in plasma and lung tissue, while mice with an ACE2 knockout genetic background developed more severe lung injury (Imai et al., 2005). In another study, mice infected with H7N9 influenza virus were found to have increased Ang II levels accompanied by decreased ACE2 protein expression in the lung tissue after 3 days (Yang et al., 2014). The enzymatic cleavage of Ang II by ACE2 produces Ang 1–7, which is in itself biologically active and initiates protective effects through the Mas receptor (Santos et al., 2018). Ang 1–7 has been recognized as the principal mediator of the beneficial effects of recombinant ACE2 in a murine model of Ang II-mediated myocardial fibrosis (Patel et al., 2015) and as a protective treatment against ARDS induced by bronchial acid instillation and high stretch ventilation in rats (Zambelli et al., 2015).

The role of ACE activity in VILI is somewhat controversial and may depend on the exact parameters of the model system, the biological compartments that were investigated, and the analytic methods. In mechanically ventilated rats with tidal volumes of 18 ml/kg aiming at moderate alveolar hyperdistension, lung ACE activity was significantly decreased (Behnia et al., 1996). In a more recent study, an imbalance between ACE and ACE2 activity was found in ventilated rats exposed to lipopolysaccharide (LPS), resulting in increased Ang II and reduced Ang 1–7 levels in BALF (Wosten-van Asperen et al., 2011). In this model, the proinflammatory effects were attenuated by treatment with losartan or cyclic Ang 1–7. In another study using a lung injury model induced by instillation of hydrochloric acid and increased tidal volumes of 18 ml/kg in rats, infusion of Ang 1–7 improved oxygenation and reduced inflammation in the acute setting and led to reduced pulmonary collagen deposition after 2 weeks if it was continued through osmotic minipumps (Zambelli et al., 2015). Plasminogen activator inhibitor (PAI)-1 is a biomarker of ARDS (Bhargava and Wendt, 2012) and was increased in conjunction with Ang II in a rat model of VILI (Chen et al., 2008). Systemic PAI-1 levels and VILI were attenuated by treatment with the ACE inhibitor captopril, and, interestingly, also hepatic ischemia/reperfusion-induced lung injury in rats was ameliorated by treatment with captopril (El-Sayed et al., 2020).

Age is an important factor for interpreting the results of animal lung injury models. In a systematic review, Schouten et al. (2015) noted that older animals developed more edema, a higher degree of histological pulmonary damage, and were found to have higher mortality than juvenile/adult animals in studies of VILI, pneumonia, and lung injury induced by hyperoxia or LPS. In a study in rats, the same group found higher wet/dry lung weight ratio, BALF protein content, and proinflammatory cytokine levels in older animals exposed to VILI (tidal volume 15 mL/kg) and LPS (Schouten et al., 2016). The same study also investigated ACE and ACE2 and found that treatment with LPS alone or in combination with injurious mechanical ventilation led to an age-dependent decrease in membrane-bound ACE activity in lung tissue and to an increase in soluble ACE activity in BALF. Levels of soluble ACE activity correlated well with indicators of lung injury severity. The study also discussed a link between increased levels of tumor necrosis factor-α in BALF and subsequent activation of ADAM9, one of the enzymes responsible for ACE shedding. In addition to lung injury, Ang II promotes monocyte/macrophage infiltration in other tissues, reactive oxygen species production, and ageing-related neurodegeneration (Benigni et al., 2010).

Expression levels of RAS enzymes seem to depend on partial pressure of oxygen. Experimental models in mice (FiO_2_ = 12%) as well as primary murine alveolar epithelial type II cells and human small airway epithelial cells exposed to 1% oxygen have demonstrated a hypoxia-induced increase in ACE2 mRNA expression (Sturrock et al., 2021). Furthermore, a cell culture study with primary murine pulmonary endothelial cells showed even higher ACE2 mRNA expression after exposure to hyperoxia (95% oxygen) and oscillating oxygen conditions between 0 and 95% than with hypoxia (5% oxygen) (Wohlrab et al., 2021). Both hypoxia and hyperoxia may occur in the lungs of patients with ARDS depending on regional ventilation.

## Clinical Evidence About the Role of the Renin-Angiotensin System in Acute Respiratory Distress Syndrome

A beneficial effect of ACE inhibitor intake in patients was demonstrated for the first time by Mortensen et al. (2005) in hospitalized patients with community-acquired pneumonia. In this study, 30-day mortality was lower in patients with previous ACE inhibitor treatment. In a population-based study, the 90-day risk for hospitalization with pneumonia was also reduced in patients over 65 years of age with a new prescription of antihypertensive drugs if the prescribed drugs were ACE inhibitors of angiotensin receptor blockers (Shah et al., 2014). Another retrospective study addressed the impact of preexisting ACE inhibitor or angiotensin receptor blocker therapy during intensive care unit (ICU) admission on the course of ARDS (Kim et al., 2017). Although patients taking a RAS inhibitor required a longer duration of mechanical ventilation and a longer ICU stay, their survival was improved compared to patients who were not taking a RAS inhibitor. Table 1 summarizes the literature on RAS-modifying drugs and risk and outcomes of pneumonia, ARDS and radiation pneumonitis, RAS activation in critical illness, ARDS and COVID-19, and randomized controlled clinical trials targeting the RAS in these conditions.

**TABLE 1:** Clinical data about effects of RAS-modifying drugs on outcome, characterization of RAS activation, and randomized clinical trials with RAS-modifying drugs in pneumonia, critical illness, ARDS, COVID-19 and radiation pneumonitis.

Authors and year	Type of study	Collective/cohort/eligibility for inclusion	No. of participants	Treatment groups/main outcomes
**RAS modifying drugs and outcome**				
Mortensen et al., 2005	Retrospective cohort study	Patients hospitalized with community acquired pneumonia	*n* = 787	ACEi therapy/30-day mortality ↓
Caldeira et al., 2012	Systematic review and meta-analysis	Studies including patients taking ACEi or ARB and analyzing risk and mortality of pneumonia	29 studies	ACEi or ARB therapy/Incidence of pneumonia ↓ with ACEi Pneumonia related mortality ↓ with ACEi and ARB
Shah et al., 2014	Population based study	New prescription of antihypertensive drugs in patients >65 years	*n* = 254.485	Drugs: ACEi, ARB, calcium channel blockers, beta blockers and thiazide diuretics/90-day risk of hospitalization with pneumonia ↓ with ACEi and ARB
Kim et al., 2017	Retrospective case control study	Patients admitted to the ICU with ARDS	*n* = 182	ACEi or ARB therapy/With RAS inhibitor: ICU mortality ↓ Duration of mechanical ventilation ↑ Length of ICU stay↑
Sun et al., 2018	Meta-analysis	Lung cancer patients with radiation therapy	*n* = 1.412	ACEi and ARB therapy/Incidence of symptomatic radiation pneumonitis ↓ with ACEi
**RAS activation in critical illness and ARDS**				
Gleeson et al., 2019	Prospective observational study	Patients admitted to the ICU	*n* = 20	Renin in plasma
Reddy et al., 2019	Pilot study, observational study	ICU patients with ARDS	*n* = 39	Angiotensin metabolite profile in plasma, protease inhibited
Annoni et al., 2019	Observational study	Mechanically ventilated ICU patients with ARDS	*n* = 96	ACE and ACE2 protein levels in plasma
Krenn et al., 2020	Observational study	Mechanically ventilated ICU patients with ARDS	*n* = 27	Angiotensin metabolite profile in RAS equilibrium analysis, active ACE levels, ACE and ACE2 protein levels in plasma
Gerard et al., 2021	Retrospective study	Patients with COVID-19 related/non-COVID ARDS	Tissue: COVID *n* = 15/non-COVID *n* = 13, Serum: COVID *n* = 35/non-COVID *n* = 24	ACE and ACE2 protein expression in lung tissue Ang II, Ang 1–7, ACE and ACE2 protein levels in serum
**RAS activation in COVID-19**				
Ozkan et al., 2021	Observational study	Hospitalized patients with COVID-19 and signs of pneumonia	*n* = 112	Ang II levels in serum
Kutz et al., 2021	Observational study with matched controls	Patients with COVID-19 admitted to the hospital and SARS-CoV-2 negative propensity-score matched controls	*n* = 43 in both groups	Angiotensin metabolite profile in RAS equilibrium analysis, angiotensin concentration-based markers of renin, ACE and ACE2 activities
Files et al., 2021	Observational study	Patients with moderate to severe acute respiratory failure due to COVID-19 and moderate acute respiratory failure negative for SARS-CoV-2	COVID-19: *n* = 22, SARS-COV-2 negative patients: *n* = 11	Ang II and Ang 1–7 plasma levels and ACE, ACE2 and POP activities in serum in fluorescent assays
Eleuteri et al., 2021	Observational study	Patients with COVID-19 related ARDS admitted to the ICU	*n* = 32	Renin, Ang I, Ang II, Ang 1–7 serum levels, Ang I/Ang II ratio as indicator of ACE activity
Reindl-Schwaighofer et al., 2021	Observational study	Patients hospitalized with COVID-19, and critically ill patients with influenza pneumonia	COVID-19: *n* = 126 Influenza: *n* = 27	Equilibrium plasma levels of Ang II, Ang 1–7 and active ACE2 levels
Wang et al., 2021	Observational study	Patients hospitalized with COVID-19	*n* = 242	Circulating Ang I, Ang II, Ang 1–7 and ACE2 levels
Osman et al., 2021	Observational study	Patients hospitalized with COVID-19 and healthy SARS-CoV-2 negative controls	COVID-19: *n* = 44 (16.6% male) Controls: *n* = 15 (46.7% male)	Ang I, Ang II, Ang 1–7 and ACE2 plasma levels, ACE2 mRNA expression and ACE2 and specific biomarker membrane protein expression in flow cytometry in peripheral blood mononuclear cells
**RAS modifying drugs in randomized controlled clinical trials**				
Boldt et al., 1995	RCT	Critically ill patients	*n* = 45	Enalapril/Hemodynamic and respiratory parameters
Khan et al., 2017	RCT	Patients with ARDS	*n* = 46	rhACE2/PaO_2_/FiO_2_ ratio, biomarkers, SOFA score
Small et al., 2018	RCT	Lung cancer patients with radiation therapy	*n* = 33	Captopril/Incidence of symptomatic radiation pneumonitis ↓
Sio et al., 2019	RCT	Lung cancer patients with chemo-radiation therapy	*n* = 23	Lisinopril/Incidence of chemo-radiation induced pulmonary distress ↓

*ACE, angiotensin converting enzyme; ACEi, ACE inhibitor; Ang, angiotensin; ARB, angiotensin receptor blocker; ARDS, acute respiratory distress syndrome; ICU, intensive care unit; RAS, Renin-angiotensin system; RCT, randomized controlled trial; rhACE2, recombinant human ACE2; POP, prolyl oligopeptidase, SOFA score, sequential organ failure assessment score.*

Actions of Ang II in ARDS are proinflammatory, profibrotic (Hrenak and Simko, 2020) and involved in regulation of alveolar fluid clearance in the lung (Deng et al., 2012a,b). It can therefore be speculated that RAS activation may be a relevant parameter in the pathogenesis and course of ARDS. Biomarker phenotyping of patients with ARDS has yielded at least two classes, including a “reactive” phenotype with higher proinflammatory activation associated with higher mortality (Calfee et al., 2014, 2015; Bos et al., 2017). Considering the association of increases in Ang II with proinflammatory activation in experimental lung injury (Jerng et al., 2007), it would be interesting to explore whether this phenotype is associated with higher RAS activation as well.

**FIGURE 2:**
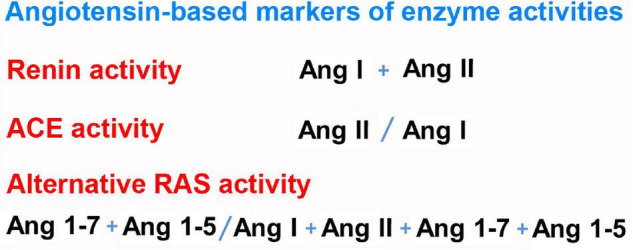
Definitions of angiotensin-based markers of renin-angiotensin system enzyme activities.

**FIGURE 3:**
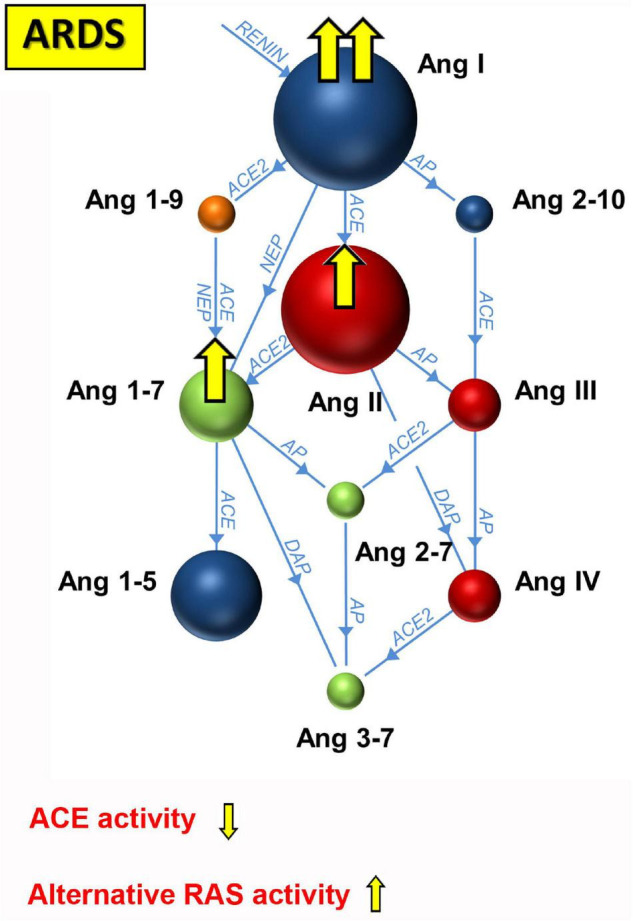
Changes in the angiotensin metabolite profile in plasma of patients with ARDS. Studies in patients with ARDS revealed increased renin-angiotensin system (RAS) activation with higher Ang I than Ang II concentrations and increased Ang 1–7 levels. Decreased Ang II/Ang I ratios and increased alternative RAS activation may be caused be decreased ACE and increased ACE2 activities, or other, yet-to-be characterized, enzymes that process Ang II.

So far, few studies have systematically examined RAS activation in mechanically ventilated ICU patients. One study measured the plasma renin levels of 20 critically ill patients at multiple time points, and found an inverse correlation with urine output and mean arterial blood pressure (Gleeson et al., 2019). As a marker of tissue perfusion, renin outperformed lactate levels in prediction of prognosis in this study. Nearly two thirds of the included patients were in septic, hemorrhagic, or cardiogenic shock, but only two patients had a diagnosis of pneumonia, so that no conclusions about renin levels in ARDS can be drawn from this study. The angiotensin metabolite profile in mechanically ventilated patients with ARDS was investigated by Reddy et al. (2019) with mass spectrometry in protease-inhibited samples, and by Krenn et al. (2020) with RAS equilibrium analysis without protease inhibition. An analysis of plasma from blood samples that are immediately stabilized with a protease inhibitor cocktail yields the circulating concentrations of angiotensin metabolites. In contrast, RAS equilibrium analysis uses plasma or serum without protease inhibition. Before measurement with liquid chromatography tandem mass spectrometry (LC-MS/MS), the samples are incubated at 37°C to establish an equilibrium between production and cleavage of angiotensin metabolites. In serum or plasma, this is feasible due to the high molar surplus of angiotensinogen to renin that is physiologically present and the constant formation of the initial substrate Ang I of the RAS cascade during the 37°C incubation step. Nascent Ang I is immediately converted into downstream metabolites by soluble enzymes so that new equilibrium levels emerge. These levels arise from equal enzymatic formation and degradation rates of individual angiotensin metabolites in the sample and depend on all enzymes involved in the plasma angiotensin metabolism, so that ratios between product and precursor may be used as angiotensin-based markers of enzyme activities (Burrello et al., 2020; Zoufaly et al., 2020). Furthermore, it has been shown that the sum of Ang I and Ang II (PRA-S) is highly correlated with plasma renin activity (Goppner et al., 2019; Burrello et al., 2020; Krenn et al., 2020). A summary of angiotensin-based markers of RAS enzyme activities is presented in Figure 2. Overall, angiotensin metabolite concentrations in protease-inhibited samples show a strong correlation with their equilibrium levels (Basu et al., 2017). In the study by Reddy et al. (2019) Ang I (1–10) levels were higher in non-survivors than in survivors of ARDS, and the Ang II/Ang I ratio was lower in non-survivors than in survivors in both studies (Krenn et al., 2020). In comparison to postoperative patients, Ang I, Ang II, and Ang 1–7 plasma concentrations were increased in early ARDS, and the Ang II/Ang I ratio was inverted (Krenn et al., 2020). Figure 3 illustrates the main changes in the plasmatic angiotensin metabolite profile associated with ARDS. These results pointed to endogenous ACE inhibition, as none of the patients with early ARDS were receiving RAS-blocking drugs, and active ACE concentrations measured in a mass spectrometry-based assay and ACE protein levels measured by ELISA were not changed. After 7 days of mechanical ventilation, RAS activation indicated by PRA-S as a marker of plasma renin activity correlated with driving pressure (Krenn et al., 2020), indicating that improvement of the respiratory situation with decreased driving pressure was associated with a larger decrease in RAS activation. The relatively low Ang II levels in contrast to Ang I concentrations may have been attributable to the following causes: Part of Ang II was metabolized to Ang 1–7 by ACE2, as our ACE2 inhibition experiments suggested (Krenn et al., 2020), but other proteases may have played a role as well. Annoni et al. (2019) actually found increased ACE protein levels in mechanically ventilated patients with early ARDS who did not survive, while ACE2 protein levels had not changed. This is at least further proof that the reduced Ang II/Ang I ratios in ARDS are more likely attributable to endogenous ACE inhibition, increased metabolization of Ang II by ACE2, or other, yet unknown, proteases than to reduced ACE expression. However, using another brand of ACE and ACE2 ELISAs, Gerard et al. (2021) found decreased ACE and increased ACE2 protein levels in the serum of patients with COVID-19-related and non-COVID ARDS. These seemingly conflicting results may be due to rather small sample sizes, a mix of underlying pathologies and biomarker phenotypes in patients with non-COVID ARDS and different specificities of ELISA kits.

Novel methods to measure active ACE and ACE2 concentrations offer new opportunities to study the role of these enzymes in a clinical context. These assays work by spiking the natural substrates to the samples to determine the specific product formation rate. For analysis of ACE activity, the samples are spiked with Ang I and incubated in the presence and absence of an ACE inhibitor, while the unspecific degradation of the substrate (Ang I) and product (Ang II) is prevented with protease inhibitors. Ang II is quantified by LC-MS/MS and the specific activity of ACE is calculated by determining the inhibitor-sensitive fraction of Ang II formation. The active ACE concentration is then calculated by relating the ACE activity in the sample to the activity of recombinant human ACE in plasma. The assay to determine active ACE2 follows an equivalent process, namely spiking Ang II, application of the ACE2-specific inhibitor MLN-4760, and quantification of Ang 1–7. The Ang 1–7 formation rate is calibrated to a standard curve of ACE2 from healthy human control plasma. With the help of this method, it was discovered that high concentrations of active ACE2 in the plasma of hospitalized patients with COVID-19 depend on disease severity (Reindl-Schwaighofer et al., 2021). Increased ACE2 protein expression in the lung tissue has recently been shown in patients with ARDS caused by COVID-19 as well as ARDS caused by other reasons and was primarily located in endothelial cells (Gerard et al., 2021). In this study, the pulmonary ACE protein expression was diminished in COVID-19-related and non-COVID ARDS. The same tendency of changes with increased ACE2 and decreased ACE protein levels, paired with increased Ang 1–7 levels, were found in the serum of patients with COVID-19-related and non-COVID ARDS. This is actually the opposite to findings of decreased ACE2 expression in rodent models of lung injury (Jerng et al., 2007; Wosten-van Asperen et al., 2011), but regulation of ACE2 expression appears to differ among species (Sajuthi et al., 2020; Ziegler et al., 2020). Another difference may be the prolonged course of COVID-19 and ARDS in critically ill patients, which may be accompanied by a dysregulated interferon response as a stimulus for ACE2 expression, as opposed to the relatively short duration of experiments modeling ARDS in animals (Gerard et al., 2021).

## The Renin-Angiotensin System in COVID-19

In light of the COVID-19 pandemic, interest in the function of the RAS in the context of ARDS has grown, as ACE2 serves as the primary cell entry receptor for SARS-CoV-2 (Walls et al., 2020; Yan et al., 2020; Zhang et al., 2020). Increased plasma levels of Ang II were reported in a small group of patients with COVID-19 in the early phase of scientific description of COVID-19 (Liu et al., 2020). In patients with H7N9 avian influenza, higher Ang II plasma levels predicted a fatal outcome (Huang et al., 2014), and in a mouse study on H7N9 infection, Ang II levels were increased and lung tissue protein expression of ACE2 was decreased after 3 days (Yang et al., 2014). Based on cell culture and animal data on SARS-CoV and SARS-CoV-2 infection, and human data of ACE2 levels in conditions with increased susceptibility to severe COVID-19, multiple reviews arrived at the conclusion that Ang II levels should be increased and ACE2 should by downregulated by SARS-CoV-2 infection (Edmonston et al., 2020; Verdecchia et al., 2020; Iwasaki et al., 2021; Triposkiadis et al., 2021). Risk factors for mortality in patients with COVID-19 include older age, male gender, hypertension, type 2 diabetes mellitus, cardiovascular disease, chronic obstructive pulmonary disease, chronic kidney disease and asthma (Aktar et al., 2021). Obesity and type 2 diabetes, especially with poor glycemic control, lead to immune cell dysfunction and foster chronic inflammatory states (Fishkin et al., 2021; Rea and Alexander, 2021). It has also been reported that ACE2 expression diminishes in advanced age and in individuals with hypertension, cardiac hypertrophy, and heart failure (Rea and Alexander, 2021). Lower ACE2 serum levels were described in patients with type 2 diabetes despite increased ACE2 expression in the pancreas and lungs as well as correlation of ACE2 serum levels with HbA1c (Elemam et al., 2021). Rising soluble ACE2 levels correlated with worsening symptoms, B-type natriuretic peptide levels and mortality in patients with heart failure (Garcia-Escobar et al., 2021), and the sum of Ang 1–7 and Ang 1–5 concentrations as marker of alternative RAS activation predicted adverse events in patients with heart failure and preserved ejection fraction (Binder et al., 2019). In a large cohort of 497 patients from the Atherosclerosis Risk in Communities Study elevated soluble ACE2 levels were associated with increased cardiac biomarkers, left ventricular hypertrophy, as well as risk for hospitalization because of heart failure, risk for cardiovascular disease events and death (Hussain et al., 2021). A similar link between increased ACE2 plasma levels and male sex as well as biomarkers of ageing, cardiovascular disease and diabetes was established in even larger cohorts of elderly patients with atrial fibrillation (Wallentin et al., 2020). Although many patients with cardiovascular diseases are treated with ACE inhibitors or angiotensin receptor blockers, no negative impact of these drugs on the risk for severe COVID-19 was found (Hippisley-Cox et al., 2020). Therefore, these drugs should only be discontinued in COVID-19 patients with hemodynamic compromise (Alexandre et al., 2020). Increased expression of ACE2 and transmembrane serine protease (TMPRSS) 2 in lung tissue together with decreased soluble ACE2 levels were also observed in patients with chronic obstructive pulmonary disease (Fliesser et al., 2021). While several patient characteristics and conditions are associated with reduced soluble ACE levels, worsening of comorbidities such as heart failure or diabetes leads to rising soluble ACE2 levels. However, there is still controversy whether increased or decreased soluble ACE2 levels are indeed a susceptibility factor for severe COVID-19 (Leow, 2020; Rahman et al., 2021).

At first glance, results from studies measuring circulating angiotensin peptides and soluble ACE2 in patients with COVID-19 do not completely fit this theoretical model of increased Ang II and downregulated ACE2 levels. A study of Ang II serum levels in a cohort of 112 patients with COVID-19 reported a decrease in Ang II levels that was more pronounced in patients with ARDS and in non-survivors (Ozkan et al., 2021). Another study reported lower Ang II equilibrium levels in hospitalized patients with COVID-19 than in propensity score matched controls negative for SARS-CoV-2 (Kutz et al., 2021). ACE and ACE2 activities were unchanged in patients with COVID-19 in this study. Other studies showed slightly decreased ACE activity in the blood of patients with moderate to severe acute respiratory failure due to COVID-19 (Files et al., 2021) and higher Ang II levels in survivors than in non-survivors of COVID-related ARDS (Eleuteri et al., 2021). This is in line with the results from a study on severe sepsis that indicated low levels of Ang II and ACE on day 1 as predictors of mortality (Zhang et al., 2014). As discussed above for non-COVID ARDS, low Ang II plasma levels, especially if they are lower than the Ang I levels, may be caused by reduced ACE activity or by increased processing of Ang II into Ang 1–7 by ACE2 (Krenn et al., 2020) or POP (Triposkiadis et al., 2021). Increased ACE2 levels in the blood of patients with COVID-19 have already been described by several studies using ELISA as well as mass spectrometry-based assays (Fagyas et al., 2021; Gerard et al., 2021; Lundstrom et al., 2021; Patel et al., 2021; Reindl-Schwaighofer et al., 2021; Wang et al., 2021)., and were higher in more severely ill patients (Fagyas et al., 2021; Patel et al., 2021; Reindl-Schwaighofer et al., 2021). An increasing trend in ACE2 plasma levels within 7 days from hospital admission indicated a higher 90-day mortality (Wang et al., 2021). Differences in soluble ACE2 levels between sexes may also play a role, as a study involving only 16.6% men within the COVID-19 group found actually lower ACE2 and higher Ang I and Ang II plasma levels in patients with COVID-19 compared to SARS-CoV-2 negative controls (Osman et al., 2021). One explanation may be that ACE2 was shed from the lung tissue after infection with SARS-CoV-2 and therefore increasingly appeared in the circulation. However, the study by Gerard et al. (2021) showed that ACE2 protein expression in lung tissue was increased in patients who died from COVID-19-related ARDS, and that the pulmonary ACE2 expression was primarily localized to endothelial cells, while the number of alveolar type II cells was reduced. Whether the lung is indeed the source of circulating ACE2 is uncertain, but interestingly, the soluble ACE2 species found in the plasma of patients with COVID-19 had specific characteristics: There was less full-length ACE2, while the 70 kD species was increased (Garcia-Ayllon et al., 2021). It remains unknown whether this has functional implications for the pulmonary endothelium. Overall, the clinical findings at present contest the hypothesis of increased Ang II and decreased ACE2 in COVID-19 as a systemic phenomenon, while these changes might well play a role locally within the lung tissue (Gerard et al., 2021; Iwasaki et al., 2021). Furthermore, the above-mentioned clinical studies are difficult to compare to each other due to several limitations. The investigated patient cohorts differed in the severity of disease, grading of ventilatory support, and time points of sampling, so that differences may have been missed, for instance between patients requiring non-invasive versus invasive mechanical ventilation. The delay from the first positive test or onset of symptoms to sampling may also vary within and between studies. The studies offering information on circulating angiotensin metabolite concentrations in patients with COVID-19 are included in Table 1. In summary, RAS activation in severe COVID-19 may be the result of a variety of changes caused by COVID-19, ARDS, and critical illness with hemodynamic instability and acute kidney injury (Zarbock et al., 2021).

Angiotensin converting enzyme polymorphisms contribute to the relatively high standard deviation of Ang II plasma levels in patient cohorts and are associated with the severity of ARDS (Pabalan et al., 2021). From the beginning of the SARS-CoV-2 pandemic it has been obvious, that disease severity varies greatly between patients spanning a spectrum from no symptoms at all to critical illness with high mortality. A likely explanation for this phenomenon may be yet unknown genetic factors that predispose patients for one or the other outcome. Possible contributors may include the known polymorphisms in the genes of the RAS enzymes ACE and ACE2. The ACE gene is located on chromosome 17q23 and exhibits an insertion/deletion (I/D) polymorphism of a 287-base pair Alu repeat sequence in intron 16, giving rise to II and DD homozygotes, respectively, and ID heterozygotes. Different genotypes vary in the expression levels and plasma activities of ACE (Rigat et al., 1990; Gard, 2010), which can also influence the responsiveness to therapeutic ACE inhibitors (Haas et al., 1998). Several studies have shown considerable association between ACE genotype (as observed with variable prevalence in different ethnic populations) and various disease endpoints such as sepsis, ARDS (Pabalan et al., 2021), and risk of pneumonia (Nie et al., 2014). In the context of COVID-19, there may also be a connection between frequency of ACE genotypes in populations and severity and outcome of this disease (El-Arif et al., 2021). Furthermore, single-nucleotide polymorphisms in the ACE2 gene on chromosome Xp22.2 have been discussed as possible predetermining factors for COVID-19 severity. The best-characterized ACE2 polymorphism is the splice region variant (rs2285666, G > A, Intron 3/4), which has also been shown to be associated with hypertension, coronary heart disease and diabetes with cerebral stroke (Mohlendick et al., 2021). Other ACE2 polymorphisms may affect ACE2-spike protein binding affinity or binding of the co-factor TMPRSS2 that is needed for viral cell entry (El-Arif et al., 2021; Suryamohan et al., 2021). In a study including hospitalized patients with COVID-19 age, high soluble ACE2 levels, a low aldosterone to renin ratio and the TMPRSS2 rs2070788 non-AA genotype were factors that independently predicted disease severity (Akin et al., 2021). Allelic variants of ACE2 differ in serum levels of soluble ACE2, which also implies a possible altered susceptibility to SARS-CoV-2 infection (Mohlendick et al., 2021).

## Potential for Clinical Application

An early randomized controlled trial on the hemodynamic and respiratory effects of enalapril in 45 critically ill patients was published in 1995 (Boldt et al., 1995). While enalapril dose-dependently decreased mean arterial pressure, the cardiac index and PaO_2_/FiO_2_ ratio were higher in patients treated with enalapril than in the control group.

After a successful phase I study of pharmacokinetics and pharmacodynamics of recombinant human (rh)ACE2 in healthy volunteers (Haschke et al., 2013), a clinical trial in patients with ARDS was terminated prematurely because the predefined effects on outcome were not achieved (Khan et al., 2017). In the phase I study, intravenous treatment with ACE2 decreased Ang II levels within 30 min of infusion. Ang 1–7 levels increased, decreased, or remained unchanged, and Ang 1–5 levels increased after all investigated doses of ACE2. Interestingly, the cardiovascular effects of ACE2 administration were absent in healthy individuals. In the phase II study, Ang II levels decreased within 12 h of rhACE2 (GSK2586881) infusion, whereas no change was observed in the placebo group. At the same time, Ang 1–7 and Ang 1–5 levels were increased in the treatment group. As in the phase I study, there were no episodes of hypotension associated with study treatment in the phase II trial. rhACE2 has also been applied in an international multicenter randomized controlled trial in hospitalized patients with COVID-19 (registered at clinicaltrials.gov as NCT04335136), but results are yet to be published. A case report on rhACE2 treatment in a patients with severe COVID-19 confirmed the intended effect of a reduction in Ang II levels during twice daily intravenous treatment with rhACE2 for 7 days (Zoufaly et al., 2020).

There is evidence that ACE inhibitors protect against radiation pneumonitis in lung cancer patients (Table 1). This includes two small randomized controlled trials (Small et al., 2018; Sio et al., 2019) and several studies analyzing the incidence of radiation pneumonitis in patients with or without ACE inhibitor or angiotensin receptor blocker therapy (Sun et al., 2018).

## Current Research Gaps and Perspectives

Experimental and clinical evidence shows that the RAS is involved in VILI and other types of pulmonary inflammation where it may offer novel therapeutic targets. Despite the increasing precision and detail of describing RAS activation with mass spectrometry-based assays, many aspects of RAS enzyme activity remain elusive. ACE inhibitors, angiotensin receptor blockers and Ang 1–7 supplementation seem to protect from VILI in experimental studies in rodents, not only by affecting levels of RAS components but also by acting on proinflammatory cytokines such as IL-6 and MIP-2, and there is evidence of decreased histological lung injury (Jerng et al., 2007; Wosten-van Asperen et al., 2011; Zambelli et al., 2015). However, clinical data suggest that decreased ACE activity indicated by a low Ang II/Ang I ratio in early ARDS in mechanically ventilated patients is a poor prognostic sign, and that this decreased Ang II/Ang I ratio likely does not come from reduced circulating ACE protein levels. One may speculate that this is an endogenous protective mechanism in patients with ARDS to limit generation of potentially harmful Ang II that is not sufficient for improvement in non-survivors. However, hypoxia only causes death in a minority of patients with ARDS (Esan et al., 2010). Thus, non-survivors of ICU-stays also include patients with multi-organ failure or acute complications such as intracranial bleeding or myocardial infarction. Reasons for the diminished Ang II/Ang I ratio in ARDS may include the presence of a yet-to-be characterized endogenous ACE inhibitor, increased ACE2 activity (Krenn et al., 2020; Reindl-Schwaighofer et al., 2021), or increased activity of other, yet-to-be characterized proteases that process Ang II. Future mechanistic investigations will have to study which molecular mechanisms are really involved. In addition, the activity levels and concentrations of ACE and ACE2 in plasma are regulated by shedding of parts of the molecules from their cells of origin in various organs. This may be the primary reason why ACE2 increases in the systemic circulation in certain disease states. The mechanisms that lead to ACE2-shedding involve proteases, e.g., tumor necrosis factor-α convertase (TACE, ADAM17) (Lambert et al., 2005). Interestingly, ADAM17 activation may be enhanced by Ang II acting on AT 1 receptors and by bradykinin (Dey et al., 2010; Rahman et al., 2021), while ACE is the most important enzyme for inactivation of bradykinin (Schmaier, 2002). Based on this mechanism, ACE inhibition may also be involved in increased shedding of ACE2. Novel assays for calculating active ACE and ACE2 concentrations will help to further study the impact of these enzymes in ARDS, but a future goal will also be to further clarify from where the enzymes are shed and by which mechanisms.

Another broad field for future study is applying the findings about involvement of the RAS in ARDS in randomized controlled clinical trials. Various trials currently listed in *clinicaltrials.gov* aim to include patients with COVID-19 and to test a treatment with angiotensin receptor blockers, ACE inhibitors, or Ang 1–7. Apart from selecting the most suitable substances, the challenge is to establish the most effective form of application. Since the lungs can be reached by circulation, systemic application of RAS-modifying drugs has a substantial impact on angiotensin metabolite concentrations in plasma (Kovarik et al., 2015; Khan et al., 2017). Inhalation therapy is another option for treating ARDS. Depending on the molecule, this method may offer the benefit of positive local effects on the bronchial/alveolar epithelium without systemic toxicity. As another advantage, inhalation of RAS-modifying drugs can be expected to have fewer effects on systemic hemodynamics. As an example, inhaled Ang 1–7 has already been studied as an anti-inflammatory therapeutic agent in a mouse model of ovalbumin-induced chronic asthma (Magalhaes et al., 2020).

## Conclusion

In summary, knowledge about RAS activation in experimental lung injury and clinical ARDS is increasing in quantity and detail. The mechanisms of regulating RAS enzyme activities and their shedding remain elusive, especially in the clinical setting, and require further study with innovative measurement tools. However, even with the current conception of RAS activation in ARDS, clinical studies can be designed to counteract primary and ventilator-induced lung injury. Several clinical trials with RAS-modifying drugs are currently underway for COVID-19, with most results still pending.

## Author Contributions

KK drafted the manuscript and prepared the figures. VT, FK, and RU assisted with literature search and revised the manuscript. FK drafted Table 1. All authors read and approved the submitted version of the manuscript.

## Conflict of Interest

The authors declare that the research was conducted in the absence of any commercial or financial relationships that could be construed as a potential conflict of interest.

## Publisher’s Note

All claims expressed in this article are solely those of the authors and do not necessarily represent those of their affiliated organizations, or those of the publisher, the editors and the reviewers. Any product that may be evaluated in this article, or claim that may be made by its manufacturer, is not guaranteed or endorsed by the publisher.
